# Responses of Reactive Oxygen Scavenging Enzymes, Proline and Malondialdehyde to Water Deficits among Six Secondary Successional Seral Species in Loess Plateau

**DOI:** 10.1371/journal.pone.0098872

**Published:** 2014-06-10

**Authors:** Feng Du, Huijun Shi, Xingchang Zhang, Xuexuan Xu

**Affiliations:** 1 Institute of soil and Water Conservation, Northwest Sci-Tech University of Agriculture and Forestry, Yangling, Shaanxi, China; 2 Institute of soil and Water Conservation, Chinese Academy of Science, Yangling, Shaanxi, China; 3 State key laboratory of soil erosion and dryland farming on Loess Plateau, Yangling, Shaanxi, China; Università della Calabria, Italy

## Abstract

Drought can impact local vegetation dynamics in a long term. In order to predict the possible successional pathway of local community under drought, the responses of some drought resistance indices of six successional seral species in the semi-arid Loss Hilly Region of China were illustrated and compared on three levels of soil water deficits along three growing months (7, 8 and 9). The results showed that: 1) the six species had significant differences in SOD, POD activities and MDA content. The rank correlations between SOD, POD activities and the successional niche positions of the six species were positive, and the correlation between MDA content and the niche positions was negative; 2) activities of SOD, CAT and POD, and content of proline and MDA had significant differences among the three months; 3) there existed significant interactions of SOD, CAT, POD activities and MDA content between months and species. With an exception, no interaction of proline was found. Proline in leaves had a general decline in reproductive month; 4) SOD, CAT, POD activities and proline content had negative correlations with MDA content. Among which, the correlation between SOD activity and MDA content was significant. The results implied that, in arid or semiarid region, the species at later successional stage tend to have strong drought resistance than those at early stage. Anti-drought indices can partially interpret the pathway of community succession in the drought impacted area. SOD activity is more distinct and important on the scope of protecting membrane damage through the scavenging of ROS on exposure to drought.

## Introduction

Drought can impact many ecosystem processes and their function, such as the vegetation succession, biodiversity, productivity and sustainability, etc [1], [2]. And drought is a most limiting factor of plant growth and reproduction worldwide [3]–[6]. Drought can intermittently disturb water cycle of ecosystem, and the carbon and mineral cycle that closely interact with water cycle [6]. These can stunt live beings indirectly through decreasing the resource availability [7]. And also, drought can hurt live beings directly and result in the decrease of their fitness, the dying off of individuals and the dying out of species at local or globe scale [6], [8]. In the past, many works had been done on the scope of drought-induced phenomena, processes and mechanismsof plants, anti-drought field practices, and breedings of drought tolerant species or varieties, as well [7], [9], [10]. Most of these works were dealt with crop plants for the purpose of food, feed and fuel products improvement. Meanwhile, as a fact, other life-support components, as grassland or forest, are more important for the maintenance and conservation of a healthy ecosystem. In recent globe climate change background, the severity and frequency of drought were reported inclined to some extent [11]. How a local ecosystem responds to that change and what is the possible changing tendency of an ecosystem, e.g., the successional pathway (or trajectory) of rangeland and forest, would make a sense of ecosystem conservation.

Ecosystem' responses to drought are the integrative results of sensitive reactions of live beings and relative sluggish responses of life-support environment. As an important part of ecosystem, the responses of vegetation to drought are the collective reflections of community species. A long term or severe drought may alter or shift the assembly and structure of community and result in degraded succession. The succession is driven by community species' eco-physical traits, such as their fitness, reproductive tragedy and their competitive ability, etc [12], [13]. Essentially, these traits root in the comparatively sensitive physical response. All of these are strongly depended on local environment factors, especially the most limiting one, e.g., the soil water in arid or semi-arid area. Two obvious sceneries that drive succession are the difference of eco-physical traits between the early successional species and the later ones [14], and the tolerance difference among co-existing species in stress-impacted area [15].

In Loess Hilly region of China, soil moisture is one of the most limiting factors that determine plant growth and productivity [16]. In most cases, soil moisture is in a water-deficit stress for plant growth in this area. This would be an important driving factor of plant community succession. As co-existing species in communities ordinarily have different drought-resistance process and ability, their responses to local soil moisture deficit and fluctuation were important for community succession pathway. And also, individuals of a species often grow in standing conditions with spatially heterogeneous soil moisture, so their anti-drought abilities have tight link with their abundance in a community. Overall, the anti-drought abilities of co-existing species and the responses of community species to water deficit can differentiate the assembly and structure of a local community, can further shape and ramified the pathway of succession. Hence, the research of anti-drought ability of successional seral species and co-existing species in a community can promote the understanding of the dynamics, causes and pathways of community succession, and help to predict the possible consequences of local environment change under globe climate change context.

Many environmental stresses exert at least part of their effects by causing oxidative damage. Consequences of oxidative stress are species-specifically related with endogenous antioxidant content. Plants' anti-drought involves a series of biological, physical and chemical processes, of which the most critical one is to detoxify radicals. These processes mainly occur in colloid and membrane interface to refrain from membrane hurt for the purpose of normal function. The anti-drought processes and materials were nearly all participated by the enzymatic system. And especially, reactive oxygen scavenging enzymes were triggered primarily when stress occurred and were more important for its function of removing reactive oxygen species (ROS) that is over produced in drought stress [3], [4], [9], [17], [18]. The enzymes have Super oxide Dismutase (SOD), Catalase (CAT), and Peroxidase (POD). These enzymes, involving redox status meditation of plant cells, are believed to be the major reliever to stress injuries in causing cellar damage [19]. Besides that, free proline, acts as an important osmoprotectant [20], its content in the leaf under stress conditions is of utmost importance for plant adaptation of environmental stress. The main cellular components susceptible to free radicals are lipids (peroxidation of unsaturated fatty acids in membranes), proteins (denaturation), carbohydrates and nucleic acids. As one of the main products when membrane is attacked by free radical, Malondialdehyde (MDA) will accumulate, and therefore it is often used as a mark of reactive oxygen damage [21].

It was believed that co-existing species' stress tolerant abilities are responsible for the community succession to some extent [22], [23]. In the past, we found that soil water of old-fields in Loess Hilly region of China had a decreasing tendency during secondary succession [24], [25]. That is to say, species in later succession stage grow in a lower soil water status as compared with those in medium or early succession stages. This research had the following expectations: 1) Later seral species have relative higher levels of reactive oxygen enzymes activities and proline content and low levels of MDA content. 2) The correlations between the activities of reactive oxygen scavenging enzymes (SOD, CAT and POD) and MDA content, and the content of proline and MDA should be negative. As reactive oxygen scavenging enzymes and osmo-protectant can both help to improve the drought resistance ability, while MDA is a mark of plants' hurt under drought.

## Materials and Methods

### 1 Experimental fields

The experiment was conducted in pot cultivation at the drought greenhouse that is affiliated to the State Key Laboratory of Soil Erosion and Dryland Farming on Loess Plateau, China. The greenhouse is functional provided with light, temperature adjustment, air-conditioner and glass rainfall proof.

### 2 Plant materials

The six seral species are *Artemisia scoparia, Setaria viridis, Artemisia sacrorum, Artemisia giraldii, Lespedeza dahurica and Bothriochloa ischaemum*. According to our previous research, their successional niche positions were −1.45, −1.51, 0.46, 1.17, 1.60 and 2.00, respectively [19]. Among of them, *A. scoparia, S. viridis* are the dominant species of early stage during secondary succession in semi-arid Loess Hilly region of China. *A. sacrorum* and *A. giraldii* are the dominant species of medium stage, and *L. dahurica* and *B. ischaemum* are later dominant species [24]. The seedlings of the six species were transplanted into plastic pots on May 10, 2012.

### 3 Experimental design

The experiment had species, water deficit and growing stage treatments in completely randomized design. The species treatment included the above six seral species. The water deficit treatment had three deficient or watering levels. The deficient levels had mild, moderate and severe water deficient stresses, equivalent to 80%, 65% and 50% of the field capacity of the pot used soil, respectively. The field capacity of the pot filled soil was measured as 21.5%, hence the watered levels were 17.2%, 14.0% and 10.75%, respectively. The growing stage treatment was represented by sampling months, which had July, August and September. Each treatment was replicated three times.

On May 9 and 10, the experimental pots (with diameter and height) were all filled with 14 kg air-dried local soil in Loess Hilly region. The total nitrogen, phosphorous and potassium of the pot filled soil were measured as 0.075, 0.052 and 1.94%; and the available nitrogen, phosphorous and potassium were 16.07, 2.71 and 109.38 mg.kg^−1^, respectively. The unavailable water content was measured as 4.56% at 15 bar. Ten seedlings of each of the six species for every water deficient levels and sampling months were lifted from local sites of Loess Hilly region. Totally 90 seedlings with largely the same size were lifted on May 11 and 12. On the middle of May, 2011, each plastic pot was transplanted into six vigorous seedlings of one of the six species. In order to assure survivorship, the rest four seedlings were used as substitute plants, which were transplanted into non-experimental pots. The seedlings in both of the two kinds of pots were well watered in the first 15 days of cultivation. The watered level was set at 85% of the field capacity (FC) of the soil, about 18.28%. On June 1st, some withered or weak seedlings were replaced with the stand-by vigorous ones that were previously transplanted in the non-experimental pots. After that, they were cultivated under well-watered condition in the greenhouse until in the middle of June. From June 20, water deficient treatment started and the watering amount of each pot was controlled using weighting method. The weighting and watering of pots were conducted every three days. On July 20, August 15 and September 10, the leaves of the six species of each water stress treatment were sampled and their physiological and biochemical indexes were assayed.

### 4 Measurement of physiological and biochemical indexes and methods

Activities of SOD, CAT and POD, content of proline and MDA were assayed according to the references of Gao [26] (SOD and CAT) and Li [27] (POD). The activity of SOD was measured using the method of photochemical reduction inhibition of nitroblue tetrazolium (NBT). The Cat activity was measured as the decrease of absorbance of H_2_O_2_ at 240 nm per minute. The POD activity was measured using guaiacol oxidation method. The proline content was measured using acid ninhydrin method. The MDA content was measured using the thiobar-bituric acid method.

### 5 Statistical analysis

In this research, five physiological indices as the activities of SOD, CAT and POD, the contents of proline and MDA were measured. Data of the five indices were analyzed using general linear model by Data Processing System [28]. The analysis was three-way univariate ANOVAs with the activities of SOD, CAT and POD, the contents of Pro and MDA as the dependent variables, and months, deficient levels and species as the independent variables. Multivariate comparison was conducted using LSD method when the treatment effects were significant. In order to illustrate the preventive effects of SOD, CAT and POD activities, and proline content on the membrane lipid destruction, the correlations between MDA content and the activities of SOD, CAT and POD, and the correlation between MDA and proline content were analyzed. The correlations between the above indices and successional niche positions of the six species were also analyzed to demonstrate the changing tendency of drought resistant ability along successional succession.

## Results

### 1 Responses of SOD activities of the six species to water deficit

**Figure 1:**
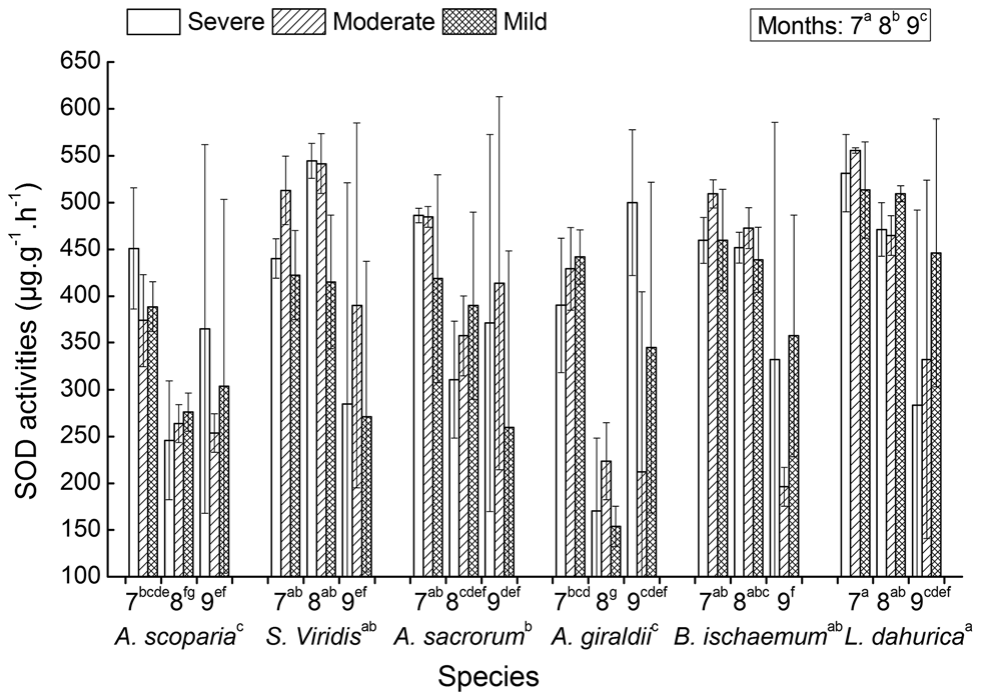
SOD activities of six successional seral species. The measurements were conducted on July (7), August (8) and September (9) under three water deficient levels (severe, moderate and mild). Significant multi-comparison results of species and month × species were marked on the second and first label rows of the X-axes, respectively (see associated ANOVAs results of Table 1).

ANOVAs showed that the six seral species had significantly different SOD activities along and among the three growing months of July, August and September (Table 1). Among of them, *L. dahurica* had highest SOD activities along the three growing months; meanwhile, *A. scoparia* and *A. giraldii* had lowest SOD activities (Fig. 1). Except *A. giraldii* had an exception, the other five species showed a monthly decreased tendency of SOD activities during the three growing season (Fig. 1). *A. giraldii* had the lowest SOD activities in August, significantly lower than the values in July and September. And also, a significant interaction effect existed between growing months and species, which implied that the six species had different changing tendency of SOD activities along the three months. For example, the multivariate comparison results showed that species of *S. viridis, L. dahurica and B. ischaemum* had significantly the lowest SOD activities in September. *A. sacrorum* had the highest SOD activities in July, significantly higher than the activities in August and September. And also, the SOD activities of *A. scoparia* in July is significantly higher than that in August, but had no significant difference with the values in September.

For the six seral species, no significant differences of SOD activities were found among the three water deficient levels (Table 1 and Fig. 1), although there existed slightly different SOD activities among the three water deficient levels. The severe, moderate and mild deficient levels had averaged SOD activities of 393.95, 388.30 and 378.44 µg.g^−1^.h^−1^, respectively. The measured SOD activities of the six species under moderate and severe water deficits had slight improvements as compared to the mild one.

**Table 1:** Responses of anti-oxidation, osmoregulation and membrane hurt of six successional seral species to three deficient levels measured on July, August and September.

Sources	df	SOD		CAT		POD		Pro		MDA	
		F	P	F	P	F	P	F	P	F	P
Month	2	19.79	1.00E-04	13.17	1.00E-04	12.53	1.00E-04	6.8	3.70E-03	11.71	1.00E-04
Deficient level	2	0.28	0.76	2.61	7.97E-02	0.57	0.56	4.14E-02	0.96	0.31	0.74
Species	5	6.85	1.00E-04	1.81	1.20E-01	6.33	1.00E-04	0.37	0.86	33.62	1.00E-04
Month × deficient level	4	0.84	0.50	4.03	4.90E-03	0.96	0.44	0.50	0.74	2.41	5.33E-02
Month × species	10	3.84	1.00E-04	10.67	1.00E-04	3.00	2.20E-03	0.28	0.98	9.07	1.00E-04
Deficient level × species	10	1.01	0.44	3.00	2.80E-03	1.27	0.25	0.52	0.86	2.63	6.70E-03
Month × deficient level × species	20	0.94	0.55	3.23	1.00E-04	1.44	0.12	0.29	9.97E-01	1.77	3.36E-02

**Figure 2:**
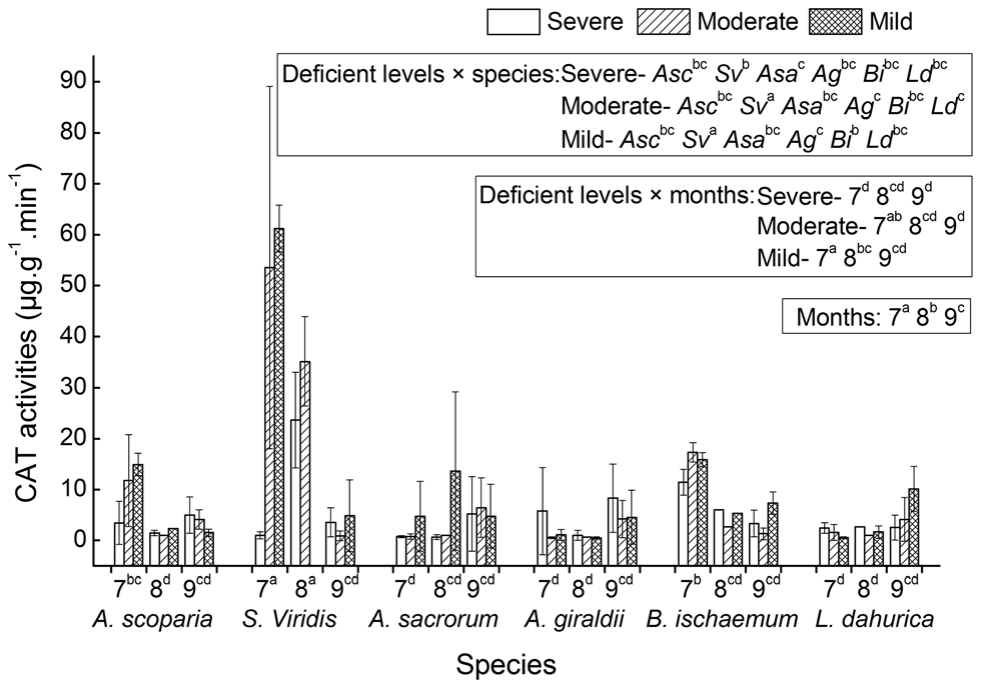
CAT activities of six successional seral species. The measurements were conducted on July (7), August (8) and September (9) under three water deficient levels (severe, moderate and mild). Significant multi-comparison results of month × species were marked on the first label row of the X-axes. And significant multi-comparison results of deficient level × species, deficient level × months and months were marked as legends within three blocks, respectively (see associated ANOVAs results of Table 1). In the box of legend zone, species of *A. scoparia, S. viridis, A. sacrorum, A. giraldii, B. ischaemum* and *L. dahurica* were abbreviated as *Asc, Sv, Asa, Ag, Bi* and *Ld*, respectively.

**Figure 3:**
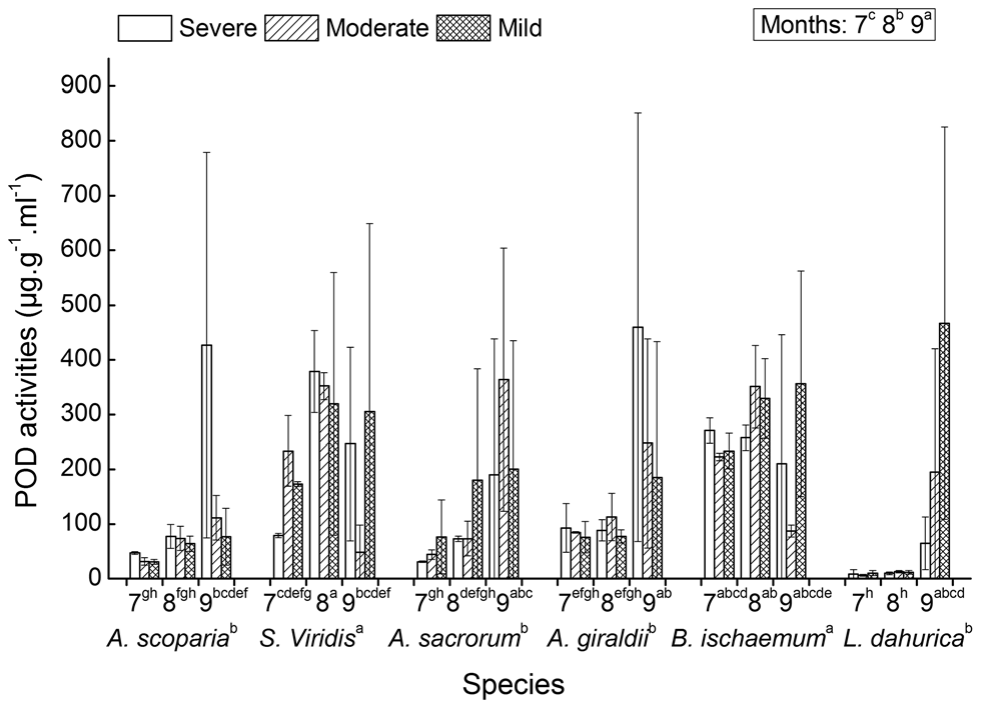
POD activities of six successional seral species. The measurements were conducted on July (7), August (8) and September (9) under three water deficient levels (severe, moderate and mild). Significant multi-comparison results of month × species and species were marked on the first and second X-axes' label rows. And significant multi-comparison results of month were marked as a legend within a block (see associated ANOVAs results of Table 1).

**Figure 4:**
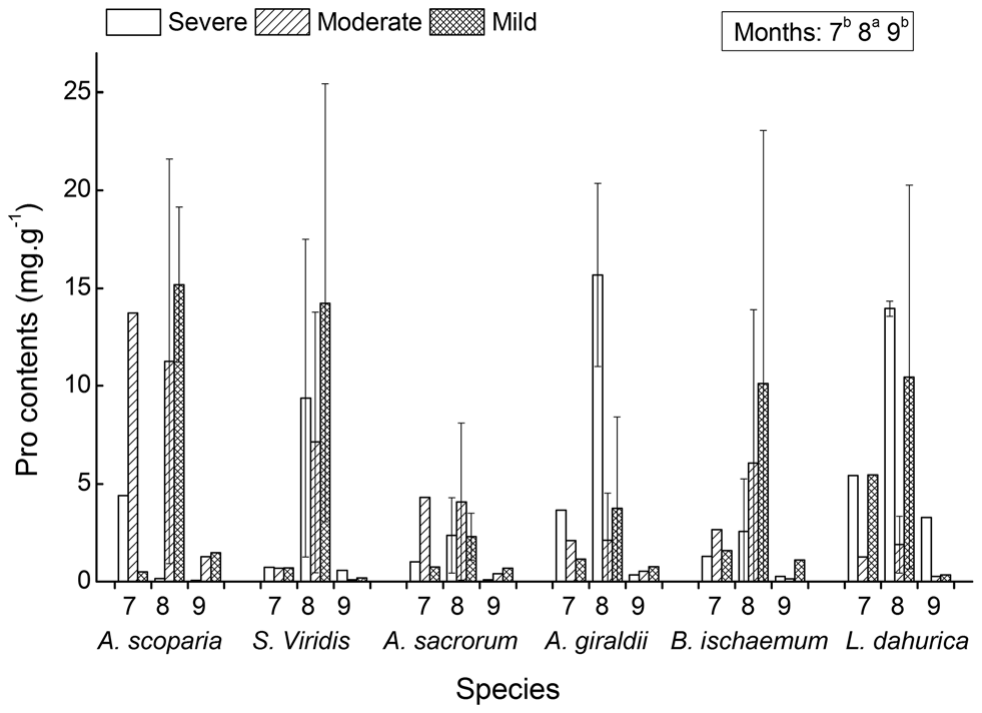
Proline contents of six successional seral species. The proline contents were measured on July (7), August (8) and September (9) under three water deficient levels (severe, moderate and mild). Significant multi-comparison results of month were marked as a legend within a block (see associated ANOVAs results of Table 1).

**Figure 5:**
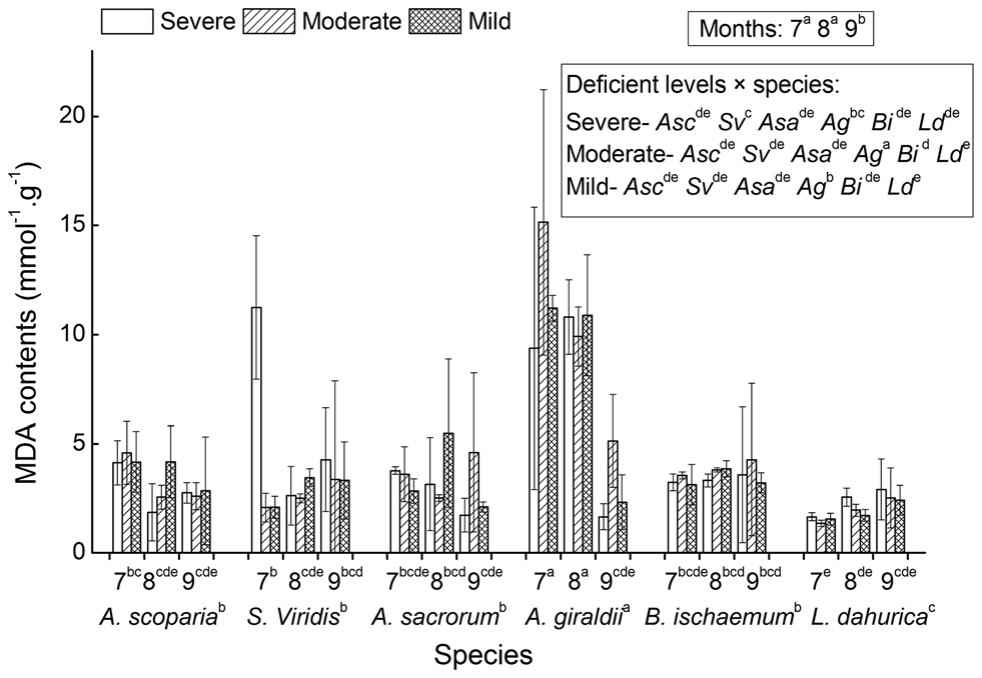
MDA contents of six successional seral species. The MDA contents were measured on July (7), August (8) and September (9) under three water deficient levels (severe, moderate and mild). Significant multi-comparison results of month × species and species were marked on the first and second label rows of X-axes. Significant multi-comparison results of month and deficient level × species were marked as legends within two blocks (see associated ANOVAs results of Table 1). In the box of legend zone, species of *A. scoparia, S. viridis, A. sacrorum, A. giraldii, B. ischaemum* and *L. dahurica* were abbreviated as *Asc, Sv, Asa, Ag, Bi* and *Ld*, respectively.

Anti-oxidation traits of the six species were measured as the activities of SOD, CAT and POD enzymes; osmoregulation and membrane hurt were measured as the contents of Pro and MDA, respectively. The table shows the results of the three-way univariate ANOVAs with the activities of SOD, CAT and POD, the contents of Pro and MDA as the dependent variables, and months, deficient levels and species as the independent variables. See Figs. 1 to 5 for means and standard errors. As the leaf samples were limited, some measurements of proline were missed, which lead to the degree freedom of total errors of proline were 83 (Fig. 4). Other items were all 161.

### 2 Responses of CAT activities of the six species to water deficit

The six seral species showed a significant decline tendency along the three months' growth (Table 1). In July, August and September, CAT activities of the six species were measured with high, medium and low levels, respectively (Fig. 2). No significant differences of CAT activities were observed among the six seral species and among the three different water deficient levels, but interaction effects of growing month × species, deficient level × species, deficient level × month and month × deficient level × species were all detected significantly. This implied that the response of CAT activities to deficient levels was a growing month related species-specific process, and the process varied with water availability.

The significant interaction between month and species was demonstrated obviously by the significant differences of *S. viridis* and *B. ischaemum* among the three growing months (Fig. 2). The differences showed that the CAT activities of *S. viridis* in September were significantly lower than the activities in July and August, and the CAT activities of *B. ischaemum* in July were significantly higher than the activities in August and September. The significant interaction between deficient level and species was illustrated by the discrepancy of CAT activities of the six species under the three water deficient levels (Fig. 2). For example, under moderate and mild deficient levels, *S. viridis* had highest CAT activities than the values of other species; while, under severe deficient level, the CAT activities of *S. viridis* had no significant differences with other species' values (except of *A. sacrorum*).

The significant interaction of CAT activities between water deficient level and growing month was illustrated by the multi-comparison discrepancy under the three water deficient levels in July, August and September (Fig. 2). Under mild and moderate deficient levels, the CAT activities of all the six species had its highest values in July; while under severe deficient level, the CAT activities had no significant difference among the three growing months.

### 3 Responses of POD activities of the six species to water deficit

The POD activities were significantly different among the six seral species and the three growing months (Table 1). *S. viridis, and B. ischaemum* had relative higher POD activities than other species had. And the POD activities showed an increase tendency along the growing season of those three months (Fig. 3). In July, August and September, the POD activities were at low, medium and the highest levels, respectively. No significant differences of POD activities were found among the water deficient levels.

The interaction effects between growing months and species were significant. The POD activities of *S. viridis* in August, and *A. sacrorum, A. giraldii, B. ischaemum and L. dahurica* in September were relative high (Table 1 and Fig. 3).

### 4 Responses of proline content of the six species to water deficit

The proline contents of the six species were found significantly different among the three months (Table 1 and Fig. 4). In August, the proline contents were significantly higher than that in July and September. No significant differences were detected among the six species and the three water deficient levels. And also, no significant interaction effects were found.

### 5 Effects of water deficit on the MDA content among the six secondary successional seral species

ANOVAs showed that MDA contents were significantly different among the three growing months and the six seral species. In September, the MDA contents were lower than that in July and August. Among the six species, *A. giraldii* had the highest MDA contents and *L. dahurica* had the lowest contents (Fig. 5). The average MDA contents under severe, moderate and mild deficient levels were 4.17, 4.23 and 3.93 mmol per gram of fresh tissue, respectively. Though MDA contents were a little higher under moderate and severe deficient levels than that under mild deficient level, they were not significantly affected by the water deficient levels (Table 1).

The significant interaction between month and species implied that the six species had their special growing time-related anti-overoxidation characteristics in terms of MDA content (Table 1 and Fig. 5). In July and August, species *A. giraldii* had the highest MDA contents, while in September, the measured MDA contents decreased to a same level with other species (Fig. 5). Though deficient levels showed no significant main effects on the MDA content, there still existed a significant interaction between deficient level and species (Table 1 and Fig. 5). For example, the MDA content of *S. viridis* under severe deficient level was significantly higher than the values under moderate and mild levels. And *A. giraldii* was measured with higher MDA content under moderate than those mild and severe levels (Fig. 5).

There also existed a significant interaction among month, deficient level and species (Table 1), which implied that the protection of membrane was a complex growing season and water status related species specific process.

## Discussion

### 1 Reactive oxygen scavenging enzymes system and osmatic adjustment play an important role in the anti-drought process

Normally, there exists a balance in plants between the production of reactive scavenging species (ROS) and quenching activity of antioxidant [29]. But when subjected to environmental adverse, the balance is upset and plants will over produce the active oxygen in excess of the capability of scavenging system, causing oxidative stress and damage. Except for some species that take avoidance or resistance tragedy when exposed to drought, most species have to tolerate drought through physiological or metabolic adaptive ways. Hence, the oxidative stress that ensues from drought is a widespread phenomenon. ROS, are viewed as main toxic cellular metabolites, are highly active molecules that can easily damage membrane and other cellular components. Therefore, on exposure to environmental stress, the scavenging of ROS is a primary process for plants' growth and reproduction. Anti-oxidation mechanisms of the cell include ROS-scavenging system and non-enzymatic antioxidants. Scavenging mechanisms for ROS involve these enzymes: SOD, CAT, POD, ascorbate peroxidase (APX) and glutathione peroxidase (GPX). Among the four major reactive oxygen species [superoxide radical O_2_^−^, hydrogen peroxide H_2_O_2_, hydroxyl radical ^.^OH and singlet oxygen ^1^O_2_], hydrogen peroxide H_2_O_2_ and the hydroxyl radical ^.^OH are mostly active toxic and destructive. Superoxide radical O_2_^−^ can be transform into H_2_O_2_ mainly through SOD or ascorbic acid. H_2_O_2_ can mainly be decomposed via the catalysis of CAT and POD.

MDA is one of the products of lipid membrane peroxidation, which can be a mark of stress-caused damage. In our case, the correlations between MDA contents and activities of SOD, CAT and POD were all negative but only the correlation of SOD and MDA was significant. That means SOD acted more effective than ascorbic acid done in the superoxide quenching, at least for the six species. The correlations between MDA contents and the above three enzymes' activities (SOD, CAT and POD) were −0.32, −0.18 and −0.058, with 0.012, 0.16 and 0.66 significant levels, respectively. The insignificant correlations between CAT, POD and MDA implied that antioxidant production does not always significantly result in the enhancement of the anti-oxidative defense. Possible reasons were: 1) Antioxidants act as a cooperative network, employing a series of redox reactions. For example, in our case, H_2_O_2_ is cooperatively scavenged by CAT and POD enzymes. Except for anti-oxidative enzymes, there also exist other non-enzymatic antioxidants that involve the membrane protection to reduce the production of MDA. 2) Other aspects as the compartmentalization of ROS formation and the localization, synthesis and transport of antioxidants would also be the determinants of the effectiveness of ROS scavenging. Some charged ROS molecule as superoxide and hydroxyl radical, they cannot cross biological membrane. 3) The important one is the ability to induce the antioxidant defense is determined by species-specific genome. The plant's response to drought is accompanied by the activation of genes involved in the perception of drought stress and in the transmission of the stress signal.

Except for oxidative stress, plants will also experience or undergo osmotic stress on exposure to drought. With the water loss of tissue, plants will accumulate a range of metabolically benign solutes, collectively known as osmolytes. Osmylytes include proline, betaines, polyols (mannitol, sorbitol, and pinitol) and trehalose, etc. Drought-caused low water potential can accelerate the degradation of structural proteins [30], from which most of osmolytes are synthesized. That is why proline content has negative correlation with relative water content (RWC) of leaves in literatures [31], [32].

The accumulation of proline can alleviate the membrane damage and reduce MDA level when plant is tolerating drought stress [33]. The observed correlation between MDA and proline content in our case was not to be an expected negative value. On the contrary it was slightly positive (pairwise Pearson = 0.039, P = 0.77). In literatures, both positive and negative correlations were reported [34]. This is probably due to: 1) The amino acid of proline is not functionally only for the preventive one of lipid membrane peroxidation. Proline involves several physiological functions and is synthesized if it is in need of these functions when plants experienced stress. These functions include membrane peroxidation prevention related group as osmotic adjustment [35], osmoprotection [36], [37], free radical scavenger activity [38], protection of macromolecules from denaturation [39], and other groups that have no direct association with the protection of membrane, as regulation of cytosolic acidity, inhibition of programmed cell death [40] and a source of accumulated carbon and nitrogen during abiotic stress. 2) And there are other ion osmotic adjustments like K^+^, Na^+^ and Cl^−^ in vacuolar, and organic osmolyte like sugar alcohols (e.g. fructans) or ammonium compound (e.g. glycine-betaine) that have the same function. 3) The compartment of proline synthesis and transport may also contribute to the ineffectiveness of membrane protection. 4) The accumulation of proline and their contribution to prevent lipid peroxidation varies among species and phonological stages [30]. For example, in our case, the six species showed different correlations between the measured proline and MDA contents. The correlations of A. *scoparia*, S. *viridis*, A. *sacrorum*, A. *giraldii*, B. *ischaemum* and L. *dahurica* were 0.36, −0.047, 0.14, 0.35, −0.12 and −0.16. The accumulation of proline mainly mediated by water potential, which is an integrative eco-physical process that is controlled by the water balance between root water absorbability and leaf water withhold. For those species with strong water absorbing and well water retention, the accumulation of proline will not be activated only when their water potential decreases dramatically. Besides that, as proline was associated with morphogenesis, it will migrate to reproductive tissues at flowering and productive period [41], [42]. Also, some authors have suggested the presence of proline protein carriers (AtProT1 and AtProT2) capable of removing the proline synthesized in the leaf and root to the reproductive organs [43]. The six seral species are in flowering and productive phase in september. Perhaps this is why the measured proline contents in leaves were relative low in September (Figure 4).

### 2 Differences of anti-drought traits of the six seral species

The activity improvement of reactive oxygen scavenging enzymes and the accumulation of osmolytes are common in many plant species in response to drought stress. Oxidative stress induced by drought often lead to the lipid membrane peroxidation. Consequently, SOD, CAT, POD, Pro and MDA are often used as important indices for evaluating the cellular redox balance and the anti-oxidative abilities. High values of SOD, CAT and POD activities and proline content, and low MDA content are the reflection of a species with high anti-drought ability. According to the responses of the five indices to water deficits (Table 1, Fig. 1–5), the drought resistant abilities of the six species can be ordinated and the adaptive responsive pattern to drought can be illustrated at large. The significant differences of SOD, POD and MDA activities among the six species plus their mean value have the following ranks of drought resistance: SOD (µg.g^−1^.h^−1^) – *L. dahurica* (456.51), *S. viridis* (424.80), *B. ischaemum* (408.70), *A. sacrorum* (388.12), *A. scoparia* (324.12) and *A. giraldii* (318.57); POD (µg.g^−1^.ml^−1^) – *B. ischaemum* (257.55), *S. viridis* (237.37), *A. giraldii* (158.27), *A. sacrorum* (136.99), *A. scoparia* (104.41) and *L. dahurica* (87.28); MDA (mmol.g^−1^) – *L. dahurica* (2.07), *A. scoparia* (3.30), *A. sacrorum* (3.32), *B. ischaemum* (3.56), *S. viridis* (3.88) and *A. giraldii* (8.50). Except *L. dahurica*, whose ranking position of POD had obvious inconsistency with SOD and MDA, the ranks of drought resistance deduced from the three items of SOD, POD activities and MDA content are roughly coincided. The ranks can interpret partially at which successional niche position will the six seral species dominate or get their highest abundances during secondary succession in Loess Hilly Plateau. The rank correlations between the deduced ranks and successional niche positions were 0.14, 0.086 and −0.14 (spearman, pairwise), respectively. This implied that species at later successional stage tend to have high SOD and Cat activity than those at early stage, and species at later stage have relative good anti-peroxidation ability than species at early stage have.

The SOD and CAT activities had a decreasing tendency (Fig. 1 and 2), whereas POD activity tend to incline at later growth period (Fig. 3). These suggest that the quenching of ROS is a kind of growth period associated synergistic function. The significant interaction between growing months and species implied that the change of anti-oxidative enzymes is species-specific phenological seasonal cycle (Table 1 and Fig. 1–3). Normally, plants have evolved seasonal cycle of physiological activity, which are phenological season related and succeed from long term's acclimation to local habitats. The anti-oxidative enzymes and osmolytes, as a part of habitats-depended physiological activity, will be naturally associated with metabolic activity strength and growth rate. The high activity of SOD and CAT in July may be ascribed to the high levels of metabolism and photosynthesis when light intensity and temperature reach their climax. The high levels of photosynthesis and metabolism provide ample substrate and energy for the synthesis of the two enzymes to avoid photoinhibitory. And vice versa, the quick growth and high levels of photosynthesis and metabolism need the smooth quenching of ROS when local habitats are in good conditions for photosynthesis and growth.

The impacts of water deficits on the five indices were not found significant as expected. This probably due to two reasons: the first one is the oxidative stress load from the seasonal light intensity or phonological cycle may overweight the load by water deficits; the second one is the watering or deficit levels is not enough to differentiate the responses of all the six species, although species responded variedly in terms of CAT activity and MDA content (Table 1 and Fig. 2 and 5).

### 3 The implication of stress tolerant traits of co-existing species to succession and ecosystem conservation

Finding the connections between co-existing species' physiological responses and the downstream ecological events is vital to understand the vegetation dynamics when environment is altered. The eco-physical traits and the relative competitive ability of co-existing species are two determinants that are responsible for the species assemblages and community succession. Especially, the adaptability of co-existing species to the most limiting environmental factor is more important in the determining their fitness and abundance in a community, which will impel community succession and determine the successional tendency or pathway. In the future, the accelerating globe change of climate and frequent local extreme climate events might shift species' temperature, water and nutrition niche. With these backgrounds, non-native species from adjacent areas may cross frontiers and become new elements of the biota, which will promote biological invasion [44]. Plants are the main active participants of mineral, water and carbon cycle through their absorbing, fixation and release. The potential vegetation change caused by climate change may influence the pattern and strength of local chemical elements cycle in the long term.
